# Fungi isolated from *Miscanthus* and sugarcane: biomass conversion, fungal enzymes, and hydrolysis of plant cell wall polymers

**DOI:** 10.1186/s13068-015-0221-3

**Published:** 2015-03-05

**Authors:** Prachand Shrestha, Ana B Ibáñez, Stefan Bauer, Sydney I Glassman, Timothy M Szaro, Thomas D Bruns, John W Taylor

**Affiliations:** Department of Plant and Microbial Biology, University of California, Berkeley, CA 94720-3102 USA; Energy Biosciences Institute, University of California, Berkeley, CA 94720 USA; Department of Environmental Science Policy and Management, University of California, Berkeley, CA 94720 USA

**Keywords:** Bioconversion, Biofuel, Fungi, Cellulose and hemicellulose degrading enzymes, Lignocellulose

## Abstract

**Background:**

Biofuel use is one of many means of addressing global change caused by anthropogenic release of fossil fuel carbon dioxide into Earth’s atmosphere. To make a meaningful reduction in fossil fuel use, bioethanol must be produced from the entire plant rather than only its starch or sugars. Enzymes produced by fungi constitute a significant percentage of the cost of bioethanol production from non-starch (i.e., lignocellulosic) components of energy crops and agricultural residues. We, and others, have reasoned that fungi that naturally deconstruct plant walls may provide the best enzymes for bioconversion of energy crops.

**Results:**

Previously, we have reported on the isolation of 106 fungi from decaying leaves of *Miscanthus* and sugarcane (Appl Environ Microbiol 77:5490–504, 2011). Here, we thoroughly analyze 30 of these fungi including those most often found on decaying leaves and stems of these plants, as well as four fungi chosen because they are well-studied for their plant cell wall deconstructing enzymes, for wood decay, or for genetic regulation of plant cell wall deconstruction. We extend our analysis to assess not only their ability over an 8-week period to bioconvert *Miscanthus* cell walls but also their ability to secrete total protein, to secrete enzymes with the activities of xylanases, exocellulases, endocellulases, and beta-glucosidases, and to remove specific parts of *Miscanthus* cell walls, that is, glucan, xylan, arabinan, and lignin.

**Conclusion:**

This study of fungi that bioconvert energy crops is significant because 30 fungi were studied, because the fungi were isolated from decaying energy grasses, because enzyme activity and removal of plant cell wall components were recorded in addition to biomass conversion, and because the study period was 2 months. Each of these factors make our study the most thorough to date, and we discovered fungi that are significantly superior on all counts to the most widely used, industrial bioconversion fungus, *Trichoderma reesei*. Many of the best fungi that we found are in taxonomic groups that have not been exploited for industrial bioconversion and the cultures are available from the Centraalbureau voor Schimmelcultures in Utrecht, Netherlands, for all to use.

## Background

To reduce the amount of carbon dioxide released into the atmosphere from fossil fuels that are used to power vehicles, biofuels must be made from entire plants and not just the sugars squeezed from their stems or the starch produced in their fruits [1]. This complete use of plant polysaccharide (especially cellulose) would maximize the amount of fuel recovered from each plant, thereby offsetting the fossil carbon required to farm the plants and minimizing the pressure to convert natural land to agriculture [2,3].

Production of these cellulosic biofuels requires a larger investment in more diverse enzymes to convert plant cell walls to sugars than is now needed to release sugar from starch [4]. Whereas enzymes account for 4.5% of the cost to make ethanol from cornstarch, they account for 17% to 20% of the cost to make ethanol from entire plants [5,6]. For cellulosic biofuel to compete with fossil fuels, it is estimated that the cost of enzymes must account for only 8% to 10% of the total cost, a twofold reduction from present costs [7].

In addition to cost, enzyme diversity is an issue because the plant cell wall, with its many polysaccharides, is far more complex than starch. These cell wall polysaccharides comprise cellulose, hemicellulosic polymers of xylose, arabinose and other sugars, and pectins, all of which are embedded in and surrounded by the polyphenolic macromolecular lignins [8]. To make complete use of plant cell walls, cocktails of enzymes capable of orchestrated digestion of these polymers will be needed. Currently, these enzymes come from industrial fermentation of the key biofuel fungus, *Trichoderma reesei* [9].

We, and others [4,10], have reasoned that fungi that naturally deconstruct the cell walls of sugarcane and *Miscanthus* might produce enzymes with the diversity and strength of activity best suited to bioconversion of these plants. Prior efforts by others at bioprospecting for mesophilic fungi have discovered five fungi isolated from sugarcane bagasse and wood with endoglucanase activities that compared favorably to enzymes from *T. reesei* [11], and 19 fungi selected from 74 species, cultivated from temperate French forests and tropical French Guiana forests, whose secretomes increase biomass conversion of maize bran when added to commercial *T. reesei* enzyme cocktails [12]. Plant pathogenic fungi have also been studied with the finding that many of these fungi bioconvert as well or better than *T. reesei* (for example, on xylans, species of *Mucor*, *Rhizoctonia*, and *Cylindrocarpon* were superior to *T. reesei*), and that fungi that parasitize monocots bioconvert these plants more effectively than fungi parasitizing dicots, and *vice versa* [13]. With thermophilic and thermotolerant fungi, 27 strains isolated from sugarcane bagasse provided thermostable endoglucanases and xylanases [14]. An interesting twist on bioprospecting involved inoculating sterilized switchgrass with decaying switchgrass for 10 serial repetitions, which returned 135 strains of two *Fusarium* species, *Fusarium sporotrichioides* and *Fusarium poae*, among which were producers of thermostable cellulases and xylanases [15].

In addition to bioprospecting, there has been research on discovering and analyzing enzymes from fungi other than the production strains of *T. reesei*, the latter having been subjected to strain improvement since the 1940s. For example, when 310 strains of *T. reesei* other than the industrial strain were assessed for their ability to deconstruct switchgrass, one strain was found capable of outperforming commercial enzyme preparations [16]. More commonly, researchers investigate strains of other fungal species. When the secrotome of *Fusarium verticillioides* grown on wheat straw was added to commercial *T. reesei* enzyme preparations, additional sugars were released from cellulose (glucose) and hemicelluloses (xylose, arabinose) [17]. Similarly, when *Chrysoporthe cubensis* was grown on sugarcane bagasse, a crude enzyme extract released more glucose and xylose than commercial enzyme preparations [18]. Also, *Penicillium echinulatum* grown on sugarcane bagasse [19] and *Penicillium brasiliensis* grown on sugar beet pulp [20] produced mixtures of enzymes more complex than commercial preparations and released sugars from cellulose and hemicelluloses. Other researchers have investigated thermophilic fungi, for example *Thermoascus auraticus* grown on switchgrass [21] or *Aspergillus terreus* grown on corn stover [22], finding that unimproved strains of these fungi produce enzymes that function as well as current commercial preparations and that remain active at temperatures as high as 70°C.

We have previously reported the isolation of 106 fungal species from seven *Miscanthus* fields and ten sugarcane plantations and the demonstration that eight of the fungi were, in fact, capable of deconstructing *Miscanthus* cell walls [10]. In this paper, we extend our analysis to 30 of fungi most often cultivated from decaying leaves and stems of these plants [10] (Table 1), as well as four fungi chosen because they are well-studied for their plant cell wall deconstructing enzymes (*T. reesei*), for wood decay (*Phanerochaetae chrysosporium* and *Postia placenta*), or for genetic regulation of plant cell wall deconstruction (*Neurospora crassa*). We extend our analysis to assess not only their ability over an 8-week period to bioconvert *Miscanthus* cell walls but also their ability to secrete total protein, to secrete enzymes with the activities of xylanases, exocellulases, endocellulases, and beta-glucosidases, and to remove specific parts of *Miscanthus* cell walls, that is, glucan, xylan, arabinan, and lignin.

**Table 1:** **Fungi studied with data on source plant**, **geographic location**, **GenBank ITS sequence**, **and CBS accession number**

**Culture** #	**Genebank accession** #	**CBS** #	**Taxa**	**Isolate ID**	**Collection date**	**Location**	**Country**	**GPS data**	**Host plant**
1	HQ631013	134065	*Aureobasidium* aff *pullulans*	sc8d50p14-8	1/22/09	Baton Rouge LA	USA	30 16′ 19′′ N, 91 5′ 43′′ W	*Saccharum officinarum*
2	HQ630970	136219	*Alternaria* aff *tenuissima*	MS3p_50-33	9/26/08	Urbana IL	USA	40 2′ 29′′ N, 88 13′ 28′′ W	*Miscanthus giganteus*
3	HQ631009	134111	*Bipolaris sp1*	sc9d100p9-2	1/22/09	Baton Rouge LA	USA	30 1′ 18′′ N, 90 47′ 00′′ W	*Saccharum officinarum*
4	HQ630963	134072	*Phoma* aff *herbarum*	MS5p50-9	9/26/08	Urbana IL	USA	40 2′ 31′′ N, 88 13′ 28′′ W	*Miscanthus giganteus*
5	HQ630972	134059	*Epicoccum* aff *nigrum*	MS7p50-17	9/26/08	Urbana IL	USA	40 2′ 34′′ N, 88 14′ 17′′ W	*Miscanthus giganteus*
6	HQ630999	134109	*Phoma* aff *glomerata*	sc13d50p14-6	1/22/09	Baton Rouge LA	USA	30 0′ 11′′ N, 90 44′ 34′′ W	*Saccharum officinarum*
7	HQ631008	135764	*Dothideomycete sp*	sc10d50p8-8	1/22/09	Baton Rouge LA	USA	30 1′ 16′′ N, 90 47′ 00′′ W	*Saccharum officinarum*
8	HQ630971	-o-	*Cladosporidium* aff *cladosporioides*	MS6p50-33	9/26/08	Urbana IL	USA	40 2′ 34′′ N, 88 13′ 31′′ W	*Miscanthus giganteus*
9	HQ631021	134015	*Aspergillus* aff *fumigatus*	BGd1p19-4	1/22/09	Baton Rouge LA	USA	29 44′ 2′′ N, 90 35′ 26′′ W	*Saccharum officinarum*
10	HQ631007	134110	*Penicillium* aff *minioluteum*	BGd100p3-1	1/22/09	Baton Rouge LA	USA	29 44′ 2′′ N, 90 35′ 26′′ W	*Saccharum officinarum*
11	HQ631027	134014	*Exophiala* aff *spinifera*	sc12d100p8-7	1/22/09	Baton Rouge LA	USA	30 4′ 1′′ N, 90 41′ 42′′ W	*Saccharum officinarum*
12	HQ630990	134064	*Exophiala* aff *salmonis*	MS4p_50-2	9/26/08	Urbana IL	USA	40 2′ 29′′ N, 88 13′ 30′′ W	*Miscanthus giganteus*
13	HQ630981	134062	*Microdochium* aff *bolleyi*	MS5p50-32	9/26/08	Urbana IL	USA	40 2′ 31′′ N, 88 13′ 28′′ W	*Miscanthus giganteus*
14	HQ630982	134063	*Nigrospora* aff *oryzae*	MS5p50-34	9/26/08	Urbana IL	USA	40 2′ 31′′ N, 88 13′ 28′′ W	*Miscanthus giganteus*
15	HQ630961	134044	*Arthrinium* aff *sacchari*	MSbale50-22	9/26/08	Urbana IL	USA	40 5′ 38.75′′ N, 88 14′ 3.10′′ W	*Miscanthus giganteus*
16	HQ630967	134073	*Arthrinium* aff *phaeospermum*	MS3p_50-12	9/26/08	Urbana IL	USA	40 2′ 29′′ N, 88 13′ 28′′ W	*Miscanthus giganteus*
17	HQ630973	-o-	*Cephalosporium* aff *gramineum*	MS5p50-12	9/26/08	Urbana IL	USA	40 2′ 31′′ N, 88 13′ 28′′ W	*Miscanthus giganteus*
18	HQ630978	134061	*Chloridium sp1*	MSbale50-42	9/26/08	Urbana IL	USA	40 5′ 38.75′′ N, 88 14′ 3.10′′ W	*Miscanthus giganteus*
19	HQ630974	134074	*Minimidochium sp1*	MS3p_50-45	9/26/08	Urbana IL	USA	40 2′ 29′′ N, 88 13′ 28′′ W	*Miscanthus giganteus*
20	HQ630984	135763	*Sporothrix* aff *lignivora*	MSbale50-11	9/26/08	Urbana IL	USA	40 5′ 38.75′′ N, 88 14′ 3.10′′ W	*Miscanthus giganteus*
21	HQ630968	134075	*Cordyceps* aff *bassiana*	MS3p_50-38	9/26/08	Urbana IL	USA	40 2′ 29′′ N, 88 13′ 28′′ W	*Miscanthus giganteus*
22	HQ630966	134071	*Gibberella* aff *moniliformis*	MS7p50-29	9/26/08	Urbana IL	USA	40 2′ 34′′ N, 88 14′ 17′′ W	*Miscanthus giganteus*
23	HQ630977	134060	*Gibberella* aff *avenacea*	MS7p50-6	9/26/08	Urbana IL	USA	40 2′ 34′′ N, 88 14′ 17′′ W	*Miscanthus giganteus*
24	HQ630964	134070	*Fusarium* aff *aethiopicum*	MS7p50-21	9/26/08	Urbana IL	USA	40 2′ 34′′ N, 88 14′ 17′′ W	*Miscanthus giganteus*
25	HQ630965	134066	*Fusarium* aff *proliferatum*	MS2-4	9/26/08	Urbana IL	USA	40 2′ 27′′ N, 88 13′ 27′′ W	*Miscanthus giganteus*
26	HQ630976	135762	*Fusarium* aff *equiseti*	MS6p50-29	9/26/08	Urbana IL	USA	40 2′ 34′′ N, 88 13′ 31′′ W	*Miscanthus giganteus*
27	HQ630960	134068	*Hypocrea* aff *lixii*	MS3p_50-23	9/26/08	Urbana IL	USA	40 2′ 29′′ N, 88 13′ 28′′ W	*Miscanthus giganteus*
28	HQ630962	134069	*Trichoderma* aff *spirale*	MSbale50-9	9/26/08	Urbana IL	USA	40 5′ 38.75′′ N, 88 14′ 3.10′′ W	*Miscanthus giganteus*
29	HQ630959	134067	*Hypocrea* aff *koningii*	MS5p50-7	9/26/08	Urbana IL	USA	40 2′ 31′′ N, 88 13′ 28′′ W	*Miscanthus giganteus*
30	HQ630969	134058	*Trichoderma* aff *atroviride*	MSbale50-8	9/26/08	Urbana IL	USA	40 5′ 38.75′′ N, 88 14′ 3.10′′ W	*Miscanthus giganteus*

-o-: culture lost. So no CBS number assigned.

We have found (1) that a majority of fungi tested equaled or exceeded the bioconversion abilities of native *T. reesei*, (2) that some of the best of these fungi are in Ascomycota taxa not previously explored for bioconversion, (3) that enzyme activity varies greatly over time and in magnitude among fungi, and (4) that comparing activities of the four enzymes studied here with amount of cell wall removed by fungal solid substrate fermentation suggests that there must be undetected enzyme activities in addition to the four tested here. By focusing on fungi isolated from energy grasses, by documenting total biomass conversion as well as the removal of four key plant cell wall components, and by measuring the activities of four key fungal enzymes, all over an 8-week period, ours is the most thorough examination of the potential contribution of bioprospecting to the biofuel industry. Our positive results provide a strong justification for evaluating the biofuel potential of the fungi that we report here and for further exploration to find fungi with additional, desirable traits.

## Results and discussion

### Biomass conversion

**Figure 1:**
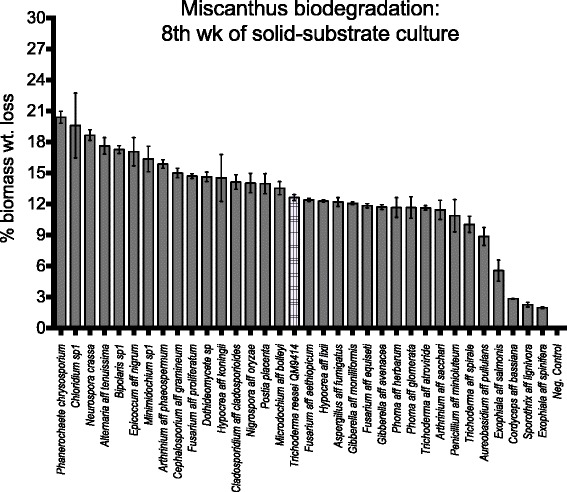
**Percent biomass** ***(Miscanthus)*** **weight reduction after 8 weeks of solid substrate cultures of fungal species on ground** ***Miscanthus.*** Performance of the industrially important enzyme producer, *Trichoderma reesei*, is shaded differently. Error bars are standard errors (*n* = 3). In addition to 30 species isolated from *Miscanthus* and sugarcane, four well-studied fungi were tested: *Phanerochaetae chrysosporium*, *Neurospora crassa*, *Postia placenta*, and the aforementioned *Trichoderma reesei*.

Over an 8-week period, we found that 25 of the 30 fungi isolated from energy grasses could convert at least 10% of *Miscanthus* biomass. Four of the five species that did not are known for growth on animals (*Exophiala* spp., *Cordyceps* sp., and *Sporothrix* sp.), and likely had been growing on animals collected along with the plant leaves (Figure 1). *Trichoderma reesei*, the industrially most important producer of bioconversion enzymes, converted 12% of the *Miscanthus*. We found 21 other Ascomycota fungi that did as well as *T. reesei*, six of which converted at least 15% *Miscanthus* (Figure 1). The most effective bioconversion fungus isolated by us was a *Chloridium* sp1, which caused 19% Miscanthus weight loss over 8 weeks, followed by *Alternaria* aff. *tenuissima* at 17%. These two fungi were significantly better than *P. placenta* and *T. reesei* and not significantly different than *P. chrysosporium* (20%), the well-studied Basidiomycota wood decay fungus, or *N. crassa* (18.7%), the Ascomycota model for studying fungal bioconversion [23] (Table 2). In total, 6 fungi isolated from decaying energy grasses were not significantly different from *P. chrysosporium*, that is, in addition to *Chloridium* sp1 and *A*. aff. *tenuissima*, *Bipolaris sp1*, *Arthrinium* aff. *phaeospermum*, *Minimidochim sp1*, and *Epicoccum* aff. *nigrum*. Several of the fungi that showed superior bioconversion were members of the Chaetosphaeriales (Sordariomycetes) and Pleosporales (Dothideomycetes), groups of fungi that have not been well-studied for bioconversion of plant biomass and that contain the very effective *Chloridium* sp1 as well as species in the genera, *Alternaria*, *Bipolaris*, and *Epicoccum*.

**Table 2:** **Matrix of Tukey**-**Kramer pairwise comparisons of % biomass reduction for all treatments**

**Fungal isolates**	***Trichoderma reesei QM9414***	***Neurospora crassa***	***Phanerochaete chrysosporium***	***Postia placenta***
*Chloridium sp1*	**6.00**	*0.99*	*0.85*	**7.42**
*Alternaria* aff *tennuissima*	**3.66**	*4.29*	*2.73*	**5.03**
*Bipolaris sp1*	*3.52*	*1.49*	*3.34*	*4.94*
*Epicoccum* aff *nigrum*	*3.30*	*1.71*	*3.55*	*4.72*
*Minimidochium sp1*	*2.56*	*2.45*	*4.30*	*3.98*
*Arthrinium* aff *phaeospermum*	*2.02*	*2.98*	*4.83*	*3.45*
*Cephalosporium* aff *gramineum*	*1.10*	*3.91*	***5.75***	*2.52*
*Fusarium* aff *proliferatum*	*0.78*	*4.23*	***6.07***	*2.20*
*Dothideomycete sp*	*0.71*	*4.30*	***6.15***	*2.13*
*Hypocrea* aff *koningii*	*0.60*	*4.40*	***6.25***	*2.02*
*Cladosporidium* aff *cladosporioides*	*0.18*	*4.83*	***6.68***	*1.60*
*Nigrospora* aff *oryzae*	*0.07*	*4.94*	***6.78***	*1.49*
*Microdochium* aff *bolleyi*	*0.46*	***5.47***	***7.32***	*0.96*
*Fusarium* aff *aethiopicum*	*1.67*	***6.68***	***8.53***	*0.25*
*Hypocrea* aff *lixii*	*1.59*	***6.07***	***7.72***	*0.32*
*Aspergillus* aff *fumigatus*	*1.88*	***6.89***	***8.74***	*0.46*
*Gibberella* aff *moniliformis*	*2.02*	***7.03***	***8.88***	*0.60*
*Fusarium* aff *equiseti*	*2.27*	***7.28***	***9.13***	*0.85*
*Gibberella* aff *avanacea*	*2.42*	***7.42***	***9.27***	*0.99*
*Phoma* aff *herbarum*	*2.45*	***7.46***	***9.31***	*1.03*
*Phoma* aff *glomerata*	*2.45*	***7.50***	***9.31***	*1.03*
*Trichoderma* aff *atroviride*	*2.49*	***7.71***	***9.34***	*1.07*
*Arthrinium* aff *sacchari*	*2.70*	***8.31***	***9.56***	*1.28*
*Penicillium* aff *minimoluteum*	*3.30*	***9.20***	***10.16***	*1.88*
*Trichoderma* aff *spirale*	*4.19*	***10.44***	***11.05***	*2.77*
*Aureobasidium* aff *pullulans*	***5.43***	***13.96***	***12.29***	*4.01*
*Exophiala* aff *salmonis*	***8.95***	***16.87***	***15.81***	***7.53***
*Cordyceps* aff *bassiana*	***11.86***	***17.48***	***18.72***	***10.44***
*Sporothrix* aff *lignivora*	***12.47***	***17.80***	***19.32***	***11.05***
*Exophiala* aff *spinifera*	***12.79***		***19.64***	***11.37***
*Phanerochaete chrysosporium*				**8.28**
*Neurospora crassa*			*1.85*	**6.43**
*Trichoderma reesei QM9414*		***5.01***	***6.86***	*1.42*

Pairwise comparisons for all treatments compared to four positive controls for ANOVA with percent weight loss at week 8 as response variable.

Legend: values in italics show no significant pairwise differences; values in bold, column is significantly lower than row; values in bold italics, column is significantly higher than row.

### Protein titers and activity profiles of cell wall degrading enzymes

**Figure 2:**
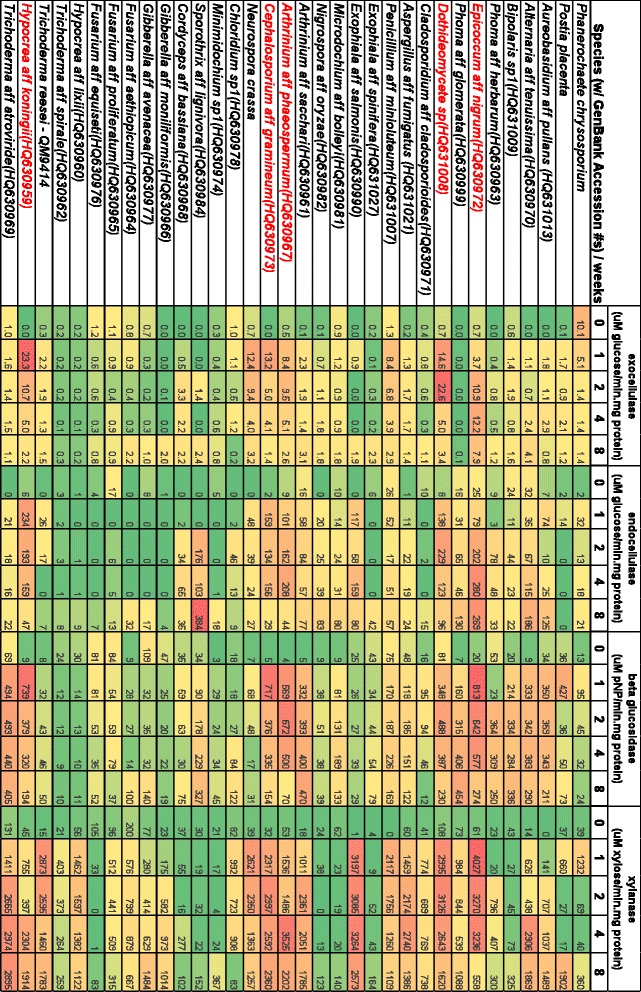
**Heat maps** **(green =** **low,** **yellow** **= intermediate** **, red =** **high)** **of enzyme activities assessed on crude-** **cell-** **free fungal extracts collected periodically from fungal cultures on** ***Miscanthus.*** GenBank accession numbers are given for fungi isolated by us from *Miscanthus* or sugarcane. Names of fungi that showed exceptional enzyme activity are shown in red.

Activities of four enzymes, exocellulase, endocellulase, beta-glucosidase, and xylanase, were measured for all 34 fungi after rehydration of lyophilized residue of solid substrate cultures that had been harvested at 0, 1, 2, 4, and 8 weeks after inoculation. Specific enzyme activities are given as μM product/min/mg protein (Figure 2) and colored as a heat map to facilitate comparison among species and time points for a single enzyme, but not among enzymes. Enzyme activity varied over an order of magnitude for exocellulase activities, over two orders of magnitude for endocellulase and β-glucosidase activities and over three orders of magnitude for xylanase activities. For all species, specific enzyme activity was minimal at time 0, and peak enzyme activity could occur at any other time point, depending on the enzyme and fungal species.

*Exocellulase activity* was highest for *Hypocrea* aff. *koningii*, 23.3 μM glucose/min/mg protein, at week 1, a level that was equaled only by *Dothideomycete* sp. at week 2 and that was twice that of any other fungus at any time. These two species and others that had substantial exocellulase activity (*P. chrysosporium*, *Penicillium* aff. *minioluteum*, *A*. aff. *phaeospermum*, *Cephalosporium* aff. *gramineum*, and *N. crassa*) reached their peak by week 2, in contrast to *E*. aff. *nigrum*, which peaked at week 4 and still showed strong activity at week 8.

**Figure 3:**
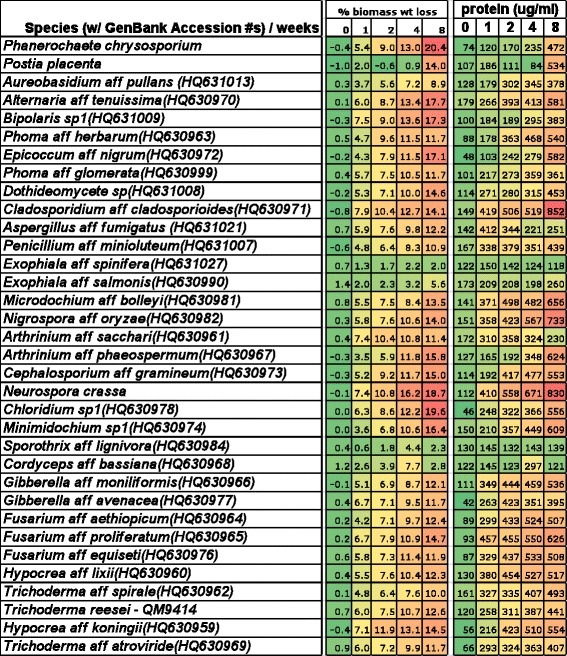
**Heat map** **(green =** **low,** **yellow** **= intermediate** **, red** **= high)** **of assessment of extracellular protein secreted by the fungi during 8 weeks of solid substrate cultures on** ***Miscanthus.*** GenBank accession numbers are given for fungi isolated by us from *Miscanthus* or sugarcane. Protein concentrations were determined using the Bradford method with bovine serum albumin as the standard.

*Endocellulase* activity showed a different pattern than exocellulase activity in that it did not peak early and then decline. Instead, most fungi with strong endocellulase activity displayed high activities at weeks 2 through 8 with the highest activities coming at weeks 4 and 8. Fungal species that had better exocellulase activities typically also had higher endocellulase activities, with the notable exception of *Sporothrix* aff. *lignivora*, which showed the highest levels of endocellulase activity seen for any of the fungi, 384 μM glucose/min/mg protein at week 8; interestingly, *S*. aff. *lignivora* lacked exocellulase and xylanase activities. Both of these anomalies are likely a consequence of the low amount of protein secreted by this animal-associated fungus (Figure 3). *E*. aff. *nigrum* again showed consistently strong activity for weeks 2, 4, and 8, and two other Dothideomycetes also achieved high levels of activity, *A*. aff. *tenuissima* and *Dothideomycete* sp. For Sordariomycetes, in addition to the aforementioned *S*. aff. *lignivora*, *A*. aff. *phaeospermum*, *C*. aff. *gramineum*, and *H*. aff. *koningii* all showed high levels of endocellulase activity over extended periods, although *H*. aff. *koningii* was unusual in having the strongest activity at week 1, 234 μM glucose/min/mg protein. Moderate to low levels of endocellulase activity were manifested by cultures of positive control species: *P. chrysosporium*, *P. placenta*, *N. crassa*, and *T. reesei QM9414*, along with *Chloridium sp1* and most species of Hypocreaceae, home to *Trichoderma*, *Gibberella*, and *Fusarium* spp., but not the aforementioned and very active, *H*. aff. *koningii*.

*Beta-glucosidase activity* showed yet a different pattern of activity, often reaching the highest level in week 1 and maintaining a high level through week 8. In addition, more species achieved the highest levels of enzyme activity for beta-glucosidase than for either exo- or endocellulases. *E*. aff. *nigrum*, *C*. aff. *gramineum*, and *H*. aff. *koningii* showed the highest activities in week 1 (813, 717, and 739 μM pNP/min/mg protein, respectively) and maintained high activities, as did *Dothideomycete* sp., and the Sordariomycete species, *Arthrinium* aff. *sacchari*, *A*. aff. *phaeospermum*, and *Trichoderma* aff. *atroviride*. The four positive control species again had moderate beta-glucosidase activities, except for *P. placenta* at the first week at 427 μM pNP/min/mg protein.

*Xylanase* activity showed a similar pattern to that of beta-glucosidase. Again, many species reached the highest levels of xylanase activity and these high levels (≥2000 μM xylose/min/ mg protein) were reached in week 1 and persisted through week 8. A difference was that the two Ascomycota control species achieved high levels of activity in the first week, *N. crassa* at 2621 μM xylose/min/mg protein and *T. reesei QM9414* at 2873 μM xylose/min/mg protein, and maintained them, but not the Basdiomycota controls, *P. chrysosporium*, and *P. placenta*. Two Dothideomycetes were exceptional, *E*. aff. *nigrum*, with the highest xylanase activity, 4027 μM xylose/min/mg protein, and *Dothideomycete* sp., at 3126 μM xylose/min/mg protein. Other fungi with high and sustained xylanase activity were the Chaetothyriales, *Exophiala* aff. *salmonis*, and the Sordariomycetes, *C*. aff. *gramineum* and *T*. aff. *atroviride*.

### Predictors for biomass loss

We analyzed relationships between biomass loss and both enzyme activity and amounts of extracellular protein. As expected, a significant relationship between the four enzyme activities and weight loss of the complex lignocellulosic substrate, *Miscanthus*, was demonstrated by regression analysis (*P* < 0.001, *R*^2^ 0.24). However, the 24% correlation between biomass loss and the four enzyme activities combined with the observation that the two best fungi for bioconversion of *Miscanthus*, *P. chrysosporium* and *Chloridium sp1*, never reached the maximum activity for any enzyme, supports the argument that most or all of the fungi studied here must harbor uncharacterized enzymes that are important to bioconversion [4]. This same conclusion has been reached by others who observed an increase in the bioconversion ability of commercial *T. reesei* enzyme preparations upon the addition of secreted proteins from a variety of fungi [11,12,24].

To determine if simple analysis of extracellular protein could predict bioconversion, we examined the correlation between the free protein titers and percent biomass weight loss. The regression model in week 8 was significant (*P* < 0.001, *R*^2^ = 0.55). The Pearson correlation is 0.7454 with *P* < 0.0001 and the scatter plot of free protein versus percent biomass weight loss at week 8 reveals a clear association between the variables. Significant correlation between protein concentration profile and percent biomass weight loss was also valid for week 2 (*P* < 0.001, *R*^2^ = 0.5) and week 4 (*P* < 0.001, *R*^2^ = 0.4035) (Figure 3).

A stepwise regression revealed that the best predictors for percent biomass weight loss at week 2 were free protein concentrations and beta-glucosidase activity. These two factors explained almost 64% of the variance present in percent biomass weight loss at week 2. These two factors were also strong predictors for percent biomass weight loss at weeks 4 and 8.

### Removal of plant cell wall components

Removal of four plant cell wall components, glucan, xylan, arabinan, and lignin, were determined from the *Miscanthus* residue that remained following 8 weeks of solid substrate cultures for the 30 fungi showing the most active bioconversion and the four fungi that are well known for bioconversion, *P. chrysosporium*, *N. crassa*, *P. placenta*, and *T. reesei*.

**Figure 4:**
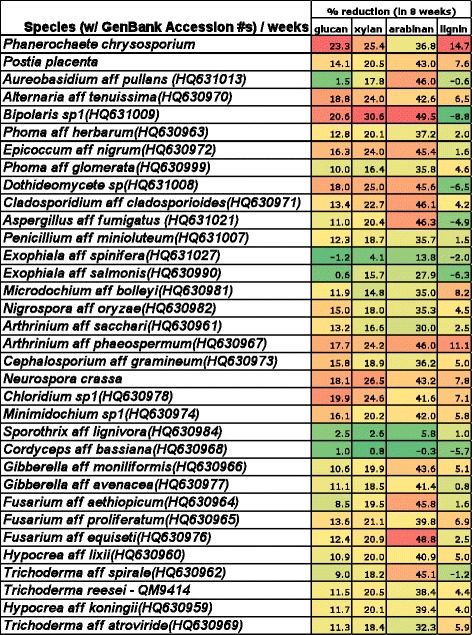
**Heat map** **(green** **= low,** **yellow** **= intermediate,** **red =** **high)** **of reduction of** ***Miscanthus*** **cell wall components by fungal bioconversion of** ***Miscanthus*** **over 8 weeks of solid substrate fermentation.** GenBank accession numbers are given for fungi isolated by us from *Miscanthus* or sugarcane.

Removal of glucan, a broad category that represents cellulose, was topped by *P. chrysosporium* at 23.3% followed by five other species with more than 18% removal, including *Chloridium* sp1 at 19.9%, *N. crassa* at 18.1%, and three Dothideomycetes. The two other positive control fungal species, *T. reesei QM9414* and *P. placenta* were able to consume 11.5% and 14.1% glucan by week 8 (Figure 4).

Removal of xylan, one of two polysaccharides representing the hemicelluloses, was the highest for *Bipolaris* sp1 (30.6%) and at or above 25% for *P. chrysosporium* (25.4%), *Dothideomycete* sp. (25.0%), and *N. crassa* (26.5%). Dropping the amount of removal to 24% included *Chloridium sp1*, *A*. aff. *phaeospermum*, and two more Dothideomycetes spp., *A*. aff. *tenuissima* and *E*. aff. *nigrum*.

Removal of arabinan, the second polymer representing hemicellulose, was almost 50% for *Bipolaris* sp1 (49.5%) and *Fusarium* aff. *equiseti* (48.8%), followed closely by eight others at more than 45% (Figure 4). The four well-studied fungi removed less than 45% of the arabinan, with *N. crassa* being the highest (43.2%) and *P. chrysosporium* the lowest (36.8%).

Lignin reduction was the highest for the well-studied, wood decay fungus, *P. chrysosporium* (14.7%) followed by *A*. aff. *phaeospermum* at 11.1%. No other fungus removed even 10% of the lignin, including the other Basidiomycota, *P. placenta* (7.6%). The best of the Ascomycota, at between 7% and 8%, were *N. crassa*, *Chloridium sp*1, *Fusarium* aff. *proliferatum*, and *Microdochium* aff. *bolleyi*, all of which were almost twice as good as *T. reesei QM9414* (4.4%). Lignin removal percentages can be difficult to measure for fungi that produce the structurally similar compound, melanin, in their cell walls. Melanin production likely is responsible for the apparent gain in lignin by the Dothideomycetes species, *E.salmonis* aff. *salmonis*, *Dothideomycete sp and Bipolaris sp1*, and the scant reduction by *Epicoccum* aff. *nigrum*.

To make rigorous comparisons of the bioconversion abilities of fungi cultivated from decaying energy grasses to that of four well-studied bioconversion fungi, *T. reesei*, *N. crassa*, *P. chrysosporium* and *P. placenta*, we performed analysis of variance (ANOVA) with percent weight loss as response variable and fungal species as the treatment (Table 2). Tukey-Kramer post hoc tests were used to determine significant difference in mean values of percent biomass weight losses and Dunn-Bonferroni adjustment and Hochberg step-up methods were used to account for Type I errors during multiple pairwise comparisons.

Mean percent weight loss varied significantly across 34 fungal treatments for week 2 (*F* = 21.62, *P* < 0.001), week 4 (*F* = 8.62, *P* < 0.001), and week 8 (*F* = 25.55, *P* < 0.001) weeks. At week 8, none of the fungal species were better at degrading Miscanthus cell walls than *P. chrysosporium* or *N. crassa*, but six were as good as *P. chrysosporium*, 15 were as good as N. crassa, 27 were as good as *T. reesei* QM9414, and 26 were as good as *P. placenta*. One newly isolated species, *Chloridium* sp1, bioconverted *Miscanthus* biomass significantly better than *T. reesei* QM9414 and *P. placenta*. The four species of animal associated fungi mentioned above, *Exophiala* aff. *salmonis*, *Cordyceps* aff. *bassiana*, *Sporothrix* aff. *lignivora* and *Exophiala* aff. *spinifera*, showed significantly lower biomass degradation.

Comparison of the amount of bioconversion at 2, 4, and 8 weeks showed that the rate of bioconversion varied by species and that the amount of bioconversion at 8 weeks was better predicted by the amount at 4 weeks than that at 2 weeks. For example, at 2 weeks, the fungi with the most bioconversion were *H*. aff. *koningii*, *Cladosporium* aff. *cladosporioides*, and *Arthrinium* aff. *sacchari*, none of which were among the top five fungal decomposers at 8 weeks. At 4 weeks, the discrepancy was not as great because the top performer, *N. crassa*, and three of the other best performers at 4 weeks, *Chloridium sp1*, *P. chrysosporium*, and *Alternaria* aff. *tenuissima*, were among the top five at 8 weeks. In fact, only one of the top five bioconversion fungi at 8 weeks was not among the top five at 4 weeks, *Minimidochium* sp1. In addition to *Minimidochium* sp1, which increased its bioconversion effort from 11.3% to 17.6% over the last 4 weeks, there were other fungi whose bioconversion increased dramatically over this period, for example, *P. placenta* increased bioconversion of *Miscanthus* from approximately 2% to 14% and *Microdochium* aff. *bolleyi* increased it from 7% to 13%.

With one exception, the fungi that best bioconverted *Miscanthus* were not among the fungi most commonly isolated from *Miscanthus* or sugarcane. The exception was *H*. aff. *koningii*, which was the fungus most commonly isolated (29%) from decaying *Miscanthus* [10]. In contrast, *Chloridium sp1* isolates comprised only 1% of fungi isolated from *Miscanthus*. Likewise, *A*. aff. *phaeospermum*, *A*. aff. *tenuissima*, *E*. aff. *nigrum*, and *Minimidochium* sp1 represented only 2.7%, 2.4%, 1.8%, and 1.2% of fungi isolated from decaying *Miscanthus* samples. With sugarcane, *Bipolaris sp1* accounted for only 1.5% of isolated strains. Had we had only conducted biomass degradation assays on the top ten species [10] associated with decaying *Miscanthus* and sugarcane, we would not have identified the more efficient biomass degradation activities of these five, moderately represented species.

Five species showed high levels of all four enzyme activities for multiple weeks: two Dothideomycetes, *E*. aff. *nigrum* and *Dothideomycete* sp., and three Sordariomycetes, *A*. aff. *phaeospermum*, *C*. aff. *gramineum*, and *H*. aff. *koningii*. This sustained activity was not seen in the positive control species, where just one activity (exocellulase for *P. chrysosporium*; beta-glucosidase for *P. placenta*, xylanase for *T. reesei QM9414*) or two activities (exocellulase and xylanase for *N. crassa*) were high during the 8 weeks.

Variation in enzyme activity over time has also been reported from other studies where the variation was seen to be as much as twofold after the first 7 days for a selected *Trichoderma* strain by Cianchetta et al. [16], twofold between days 4 and 5 for an *Aspergillus fumigatus* strain obtained from the Amazon forest [25], and twofold between the first and second weeks by strains of *A. fumigatus* and *Myceliophthora* sp. isolated from sugarcane bagasse [14]. In none of these studies was the variation as high as seen here. However, activities were monitored for four times longer in this study than in the previous studies, and, as noted above, the highest levels sometimes were achieved after the longest time interval, that is, 8 weeks.

## Conclusion

The most important conclusion from the research presented here is that wild isolates of many fungi recovered from decaying sugarcane or *Miscanthus* were capable of bioconverting ground and alkali-pretreated *Miscanthus* better than *T. reesei*, the fungus that is used to produce most of the enzymes for the commercial deconstruction of plant cell walls. In fact, 21 of the fungi tested did as well as *T. reesei*, 15 did 25% better than *T. reesei*, and one, *Chloridium sp*1 did as well as *P. chrysosporium*, the most active bioconversion fungus of the four well-studied fungi that we included as controls. Another of the four control fungi, *Neurospora crassa*, was among the best fungi at bioconverting *Miscanthus*, validating its use as a model for Ascomycota bioconversion of lignocellulose feed stock for the production of biofuels. Four of the ten best bioconversion fungi isolated from *Miscanthus* or sugarcane are in two taxa of Ascomycota with melanized mycelia, the Chaetospheriales and the Dothideomycetes. Fungi in neither of these taxa have received significant research attention in terms of bioconversion.

Regarding enzyme activity over 8 weeks, the most striking conclusion is that both the level and timing of enzyme activity are quite variable. In terms of activity, for example, xylanase varied over two orders of magnitude in activity among the fungi tested. In terms of timing, the fungi with the most active exocellulases, *Dothideomycete* sp. and *N. crassa*, reached peaks of activity early, after just 1 or 2 weeks, whereas the fungi with the strongest endocellulase activities, *Epicoccum* and *Alternaria*, reached peak activity late, at week 8. With beta-glucosidase, the period of peak activity was reached early and then maintained for a long period, for example, *T*. aff. *atroviride* and *E*. aff. *nigrum* reached peak activity in week 2 and maintained it through week 8; similarly, *H*. aff. *koningii* reached peak activity in week 1 and maintained it through week 4. For xylanase, seven species reached the highest level of activity, six doing so in the eighth week and the seventh, *N. crassa*, achieving the highest level early, at weeks 1 and 2. An important caveat in our measurements of enzyme activity is the contribution of enzyme bound to substrate, which could not contribute to our assays of enzyme activity.

In terms of the potential to discover novel enzymes useful for bioconversion of cellulosic feed stocks, based on statistical analyses, the following species are good candidates for further investigation: *Chloridium sp1*, *Epicoccum* aff. *nigrum*, *Alternaria* aff. *tenuissima*, *Bipolaris sp1*, *Arthrinium* aff. *phaeospermum*, *Minimidochium sp1*, *Cladosporidium* aff. *cladosporioides*, *Microdochium* aff. *bolleyi*, *Nigrospora* aff. *oryzae*, *Dothideomycete* sp., *Fusarium* aff. *proliferatum*, *Aspergillus* aff. *fumigatus*, *H*. aff. *koningii*, and *Cephalosporium* aff. *gramineum*. More importantly, six of these fourteen species: *Epicoccum* aff. *nigrum*, *Dothideomycete* sp., *Alternaria* aff. *tenuissima*, *Arthrinium* aff. *phaeospermum*, *Cephalosproium* aff. *gramineum*, and *H*. aff. *koningii* also showed higher levels of exo- and endocellulase, beta-glucosidase, and xylanase activities across all five time points. Two other fungi are worthy of additional research because they exhibited the highest levels of enzyme activity for at least two enzymes, *N. crassa* and *Trichoderma* aff. *atroviride*.

Another important conclusion regarding unsampled enzyme activity is that the four types of enzymes analyzed here, endocellulase, exocellulase, beta-glucosidase, and xylanase, explained just one quarter of the biomass loss; clearly, other enzymes and processes are playing important roles in biomass conversion. Two measurements explained as much as 64% of the variance in weight loss early in the *Miscanthus* fermentation, that is, at week 2, the amount of secreted protein and the beta-glucosidase activity. In fact, just the concentration of secreted protein correlated more closely with amount of biomass conversion throughout weeks 2 to 8 than summed enzyme activity, again pointing to the action of additional cell wall deconstructing enzymes.

Our final conclusions concern the removal of specific plant cell wall components, that is, glucans, xylans, arabinans, and lignin, by 14 of the best bioconversion fungi. With glucans, *T. reesei* removed less than any of the best 14 fungi. In contrast, four of the wild isolates, plus *N. crassa*, were almost as good as the best fungus, *P. chrysosporium*. With xylans and arabinans, *P. chrysosporium* and *T. reesei* were among the poorest consumers. Instead, *Bipolaris sp1* was best at removing both xylans and arabinans, followed closely by *N. crassa* and additional melanized species. When it comes to lignin, however, *P. crysosporium* is in the lead, having removed 13%, whereas no other species could remove even 10%. A confounding factor when it comes to measuring lignin removal is the production of the structurally similar compound, melanin, by some of the most active bioconversion fungi, including *Bipolaris*, *Epicoccum*, and *Alternaria*, all members of the Dothideomycetes. This production may cause an underestimation of the true amount of lignin removed.

## Methods

### Fungi

The isolation and identification of fungal isolates used in this study previously were described [10] and cultures have been deposited at Centraalbureau voor Schimmelcultures (CBS) Fungal Biodiversity Center, Utrecht, Netherlands (Table 1). The nomenclatural term, species affinis (abbreviated aff.), is used for taxa with internal transcribed spacer (ITS) sequence identities greater than 97% as compared to named species, and the term species (abbr. sp.) is used for taxa more than 3% distant from any named species.

### Substrate and pretreatment

The solid substrate for culturing was ground *Miscanthus* (20 mesh) that had been pretreated with 0.5% *w*/*v* sodium hydroxide (solid to liquid, 1:10) as previously described [10]. Following pretreatment, the *Miscanthus* was recovered via centrifugation, rinsed three times with deionized water, again recovered using centrifugation, and adjusted to pH 4.5 with sulfuric acid in the final rinse. The residue was squeezed to remove excess liquid and then air-dried at room temperature for 48 h before lyophilization and storage at −80°C.

### High throughput fungal culture tubes

*Miscanthus* bioconversion was conducted in round bottom, 15-ml polypropylene tubes [10]. Tubes were weighed, filled with approximately 600 mg pretreated *Miscanthus*, three 5 mm glass beads, and 0.5 ml deionized water, capped and autoclaved at 121°C for 20 min. To determine the initial dry weight of biomass in each tube, the tubes and contents were lyophilized, and this weight was compared to the weight of the empty tube and three 5 mm glass beads.

We chose 30 filamentous fungal isolates for our *Miscanthus* biodegradation study based on their frequency of isolation in decaying *Miscanthus* and sugarcane samples, which included some commonly and rarely isolated species, but no yeasts.

To prepare uniform inocula, fungi were grown in 100 ml of yeast malt (YM) broth as described [10,26]. Fungal colonies were fragmented in a sterile laboratory blender for 1 min and the shredded mycelium was allowed to rejuvenate for 24 h. To minimize nutrient carry over, the fungus was rinsed three times in 100 ml of aqueous NaCl (0.85%) and recovered by centrifugation at each step. Prior to inoculation, the mycelium was resuspended in 50 ml of Vogel’s medium [27] with no added sugar.

To start enough solid substrate cultures for three replicates at 0, 1, 2, 4, and 8 weeks (Figure 2) for each fungus, 15 culture tubes were inoculated with 2 ml of suspended mycelium as described [10]. The tubes were plugged with sterile foam and vortexed to mix the biomass and fungal inoculum. Vortexing also spread the mixture along the inner sides of the tube to create a space that provided for air exchange in the central axis of each tube. In addition to testing 30 fungi isolated from *Miscanthus* and sugarcane in the field, we included positive controls with four fungi known to convert biomass, *T. reesei QM9414*, *N. crassa*, *P. chrysosporium*, and *P. placenta*, and a negative control that lacked fungal inoculum. During 8 weeks of solid substrate cultures, we maintained the incubation temperature at 25°C and the relative humidity at 85 ± 5%.

### Sampling and analytical assays

We froze and lyophylized three tubes for each fungal species and controls at each sampling time (0, 1, 2, 4, and 8 weeks). Loss of biomass was calculated as the difference between the initial and final dry weights of *Miscanthus* (corrected for the dry weight of added fungal inoculum and assuming that an insignificant amount of fungal biomass was produced during bioconversion) as a percentage of the initial weight and is reported as the mean of the three tubes [10].

#### Recovery of free sugars and proteins

Following weighing, soluble sugars, organic compounds, and proteins were recovered from the lyophilized *Miscanthus* by adding 10 ml of sterile water to each culture tube, vortexing the tube for 5 min, and centrifuging the tube (2,700 × *g* for 5 min). The supernatant was then filtered (0.22 μm pore size, 25 mm GD/X PES filter membrane, catalog number 6904-2502, Whatman, Piscataway, NJ, USA) into sterile polypropylene tubes and frozen at −80°C. The residues in the culture tubes were also frozen at −80°C.

To analyze total protein (via microwell Bradford Assay) and the activities of four enzymes, xylanase, exocellulase, endocellulase, and b-glucosidase, we used a portion of the filtered, cell-free, supernatant that had been diluted (1:1) in deionized water [23].

#### Xylanase activity assay

Xylanase activity of the cell-free supernatant (50 μl) was assayed in deep 96 microwell plates with 450 μl of 1% beechwood xylan (Sigma-Aldrich, St. Louis, MO, USA), prepared as 10 g/l in 50 mM sodium acetate buffer at pH 5.0. To aid mixing and reaction, a 3 mm glass bead was added into each of the 96 wells and the sealed plate was shaken at 170 rpm for 20 h in a 37°C incubator. Controls lacked either the substrate or the cell-free supernatant. Specific xylanase activity was determined from the rate of xylose release per unit wt. of protein (μM xylose/min/mg protein) as measured by the dinitrosalicylic acid (DNS) method. The reaction supernatant was recovered by centrifugation (2,500 × *g* for 5 min) and 5 μl were added to 75 μl of DNS reagents for incubation at 99°C for 10 min. The reactions were cooled on ice and diluted with deionized water (1:3) before absorbance was measured at 540 nm. Xylose concentration was determined using a xylose standard curve prepared using xylose standards of 1, 4, 8, 10, 16, and 20 mM.

#### Exocellulase activity assay

Exocellulase activity of the cell-free supernatant (50 μl) was assayed with 450 μl of 0.5% SigmaCell 20 (Sigma-Aldrich) prepared as 5 g/l in 50 mM sodium acetate buffer at pH 5.0. The reaction conditions were same as described for the xylanase assay. Controls lacked either the substrate or the cell-free supernatant. Specific exocellulase activity was determined from the rate of glucose release per unit wt. of protein (uMglucose/min/mg protein). The reaction supernatant was recovered by centrifugation (2,500 × *g* for 5 min) and 50 μl were added to 150 μl of glucose assay solution (1.5 μl 100 mM o-dianiside, 3 μl 500 U/ml glucose oxidase, 0.3 μl 5,000 U/ml peroxidase and 145.2 μl 50 mM sodium acetate buffer) for incubation at room temperature for 45 min before absorbance was measured at 540 nm. Concentration of glucose was determined by comparison to standard curve prepared from glucose standards of 200, 400, 600, and 1,000 μM.

#### Endocellulase activity assay

Specific endocellulase activity was measured in the same manner as exocellulase with the exception that the substrate was 0.5% carboxymethyl cellulose (Sigma-Aldrich) prepared as 5 g/l in 50 mM sodium acetate buffer at pH 5.0 and that the enzyme assay plate was incubated at 37°C for 1 h. Released glucose was assayed using glucose oxidase assay as described above.

#### Beta-glucosidase activity assay

Beta-glucosidase activity of the cell-free supernatant (50 μl) was assayed with 450 μl of 500 μM *p*-nitrophenyl beta D-glucopyranoside (pNPG, Sigma-Aldrich) prepared in 50 mM sodium acetate buffer at pH 5.0. Assays were kept mixed by shaking at 170 rpm for 1 h in a 37°C incubator. Controls lacked either the substrate or the cell-free supernatant. Specific beta-glucosidase activity was determined from the rate of *p*-nitrophenol (pNP) release per unit wt of protein. The reaction supernatant was recovered by centrifugation (2,500 × *g* for 5 min) and 100 μl were mixed with 100 μl of 100 mM sodium bicarbonate before absorbance was measured at 400 nm. Concentration was determined by comparison to *p*-nitrophenol standards of 0, 10, 20, 50, 100, and 200 μM.

#### Principal biomass component analyses

To prepare biomass for analysis of the glucan, xylan, and lignin fractions remaining after solid substrate cultures, previously frozen residues were thawed and extracted four times at 65°C for 30 min each: twice in 10 ml hot water, once in 10 ml absolute ethanol, and once in 10 ml acetone. The extractive-free residue was air-dried in a chemical hood for 2 days before it was pulverized in a ball mill and dried at 105°C for 16 h. For compositional analysis, the samples were analyzed as outlined in Ibáñez and Bauer [28]. In brief, the pulverized and dried biomass (50 mg) was then incubated at room temperature with 0.5 mL of 72% sulfuric acid in a modified Hungate vial capped with a rubber stopper with vortexing every 15 min. After 1 h, 14 ml of deionized water were added, and the mixture was autoclaved for 60 min (liquid cycle, 121°C) before storage at 4°C overnight to settle the solids. Two milliliters of the clear supernatant was filtered (0.45 μm, PES) and used for high-performance liquid chromatography (HPLC) analysis at 50°C on an HPX-87H (300 × 7.8 mm, Bio-Rad, Hercules, CA, USA) column on an Agilent 1200 series liquid chromatography instrument equipped with a refractive index detector. Elution was performed with 5 mM sulfuric acid at a flow rate of 0.6 ml/min. Glucose, xylose, and arabinose (> = 99%) were obtained from Sigma-Aldrich and linearity of calibration of each standard was tested in the range of 0.01 to 20 mg/ml.

Residues that had not been digested with acid were saved for lignin and ash analyses. The lignin content was determined by the Klason method. Solids were resuspended by vortexing, then filtered through a pre-weighed glass micro filter after which both the vial, and filter were extensively rinsed with deionized water. The filter and solids were dried at 105°C overnight and weighed after cooling in a desiccator for 30 min. The solids were then ashed by incubation of the filter and content at 575°C (ramp: 105°C for 10 min, 200°C for 10 min, 300°C for 30 min, 575°C for 3 h, cooling to 105°C), cooled in a desiccator for 30 min, and weighed. The percentage of lignin was calculated as the weight of the dry solids minus that of the ash as a percentage of the weight of the initial, dry *Miscanthus* biomass.

### Statistical analyses

To compare the biomass degradation ability and extracellular enzyme activity profile of 30 fungal isolates with the four, highly studied species, mean values of the three replicates at each time point were compared. We conducted ANOVA to determine significant differences in data using percent weight loss as the response variable and fungal species as treatments. Tukey-Kramer *post hoc* tests were used to elucidate significant differences in pairwise comparisons. Corrections were made to account for type I errors and *P* values were adjusted using Dunn-Bonferroni and Hochberg step-up methods. Stepwise regressions were used to determine the variables influencing the variation in percent biomass weight loss.

## Abbreviations

ANOVA: analysis of variance
CBS: Centraalbureau voor Schimmelcultures
DNS: dinitrosalicylic acid
HPLC: high-performance liquid chromatography
ITS: internal transcribed spacer
pNPG: *p*-nitrophenyl beta D-glucopyranoside
pNP: *p*-nitrophenol
YM: yeast malt
